# Metal-Phenolic Networks Delay the Oxidation of Alkaline High-Protein Gel Foods: Improving the Quality of Coated Tofu

**DOI:** 10.3390/gels12050383

**Published:** 2026-04-30

**Authors:** Jian Zeng, Xiaohu Zhou, Yang Liu, Bing Wei, Xinrui Diao, Jie Chen, Saihua Sun, Xiangjun Li, Xuejiao Zhang, Xiaojie Zhou, Hao Chen, Zhanrui Huang, Liangzhong Zhao, Dajun Yang, Xiangle Huang

**Affiliations:** 1 Hunan Provincial Key Laboratory of Soybean Products Processing and Safety Control, Hunan Engineering Research Center of Green Processing and Equipment of Hunan-Style Food, College of Food and Chemical Engineering, Shaoyang University, Shaoyang 422000, China; foodzja@163.com (J.Z.); xinruid05@163.com (X.D.); jiechen2003@163.com (J.C.); 17353955124@163.com (S.S.); 4103@hnsyu.edu.cn (X.Z.); xiaojiezhou2020@163.com (X.Z.); spgc13@163.com (H.C.); zhanrui_huang@163.com (Z.H.); sys169@163.com (L.Z.); 2 Guangdong Provincial Key Laboratory of Aquatic Product Processing and Safety, College of Food Science and Technology, Guangdong Ocean University, Zhanjiang 524088, China; liuyang1104@126.com; 3 Research Center of Anti-aging Chinese Herbal Medicine of Anhui Province, Biology and Food Engineering School, Fuyang Normal University, Fuyang 236037, China; weibing90@fynu.edu.cn; 4 Sichuan Han Han Dou Jiang Food Co., Ltd., Chengdu 611530, China; 13548794633@163.com; 5 Hunan Qiao Da Niang Food Co., Ltd., Shaoyang 422000, China; 15573963653@163.com

**Keywords:** coated tofu, alkaline high-protein food, MPNs, antioxidant activity, protein structure, gel network

## Abstract

Under alkaline conditions, most commonly used preservatives exhibit limited efficacy and fail to meet the preservation requirements of coated tofu. This study aims to investigate the effects of metal-phenolic networks (MPNs) on quality deterioration, protein oxidation, conformation, and gel microstructure of coated tofu during cold storage (4 °C and 10 °C). The results showed that, compared with the untreated control group, MPNs treatment effectively inhibited protein oxidation, alleviated quality deterioration, delayed the degradation of color and texture, and reduced protein degradation, as evidenced by soluble protein contents that were 63.55% (4 °C) and 66.65% (10 °C) lower than those of the control group after 20 days of storage. MPNs treatment also improved the orderliness and stability of the protein secondary structure. In addition, electrophoretic analysis showed that MPNs markedly retarded the decline in band optical density of the 11S protein A subunit by 96.19% and 97.28% at 4 °C and 10 °C, respectively, and suppressed the increase in the B subunit by 13.28% and 73.20%, respectively. Moreover, MPNs treatment helped maintain a more compact gel network. Based on physicochemical characterization and Pearson correlation analysis, the preservative effect of MPNs on coated tofu under alkaline conditions was elucidated, revealing the internal correlation between the inhibition of quality deterioration and the regulation of protein oxidation. Specifically, MPNs mitigate protein disulfide bond loss, increase the β-sheet content, preserve the natural protein conformation and the relative proportion of 11S subunits, stabilize the gel microstructure, and thereby achieve quality preservation. These findings provide theoretical support and strategic reference for the development of preservation technologies for alkaline high-protein gel foods.

## 1. Introduction

Tofu is a traditional soy protein gel originating from natural plants, which is widely consumed because of its balanced nutritional properties [1]. One form of tofu is called coated tofu, which has gained rapid popularity in southern China in recent years owing to its distinctive texture of a crisp crust and a creamy, paste-like interior formed after heat treatment. Its core processing relies on alkali treatment, which degrades macromolecular substances within tofu into small-molecule components [2]. As expected, this treatment shifts the tofu from an acidic to an alkaline state (pH > 8.0). Tofu has high moisture content and poor storage stability [3]. Moreover, the alkaline environment promotes protein oxidation [4], further accelerating quality deterioration during cold storage. This often leads to loss of freshness, loosening of the protein gel network, and softened texture [5], which significantly shortens the product shelf life and limits its distribution and market expansion. Therefore, the development of suitable preservatives for coated tofu systems is of great practical importance.

Common preservatives exhibit favorable free radical scavenging activity under neutral or weakly acidic conditions, but often show poor stability in strongly alkaline environments. For instance, vitamins and enzymes such as superoxide dismutase (SOD) and catalase (CAT) may become inactivated under high alkalinity due to conformational changes in their protein structures [6,7]. Similarly, vitamin-based antioxidants, especially B vitamins, are prone to degradation under alkaline conditions [8]. Accordingly, there is an urgent need to develop composite or modified preservatives with high alkaline stability for alkaline food systems such as coated tofu. Metal–phenolic networks (MPNs) are supramolecular architectures that arise from the self-assembly between metal ions and polyphenol molecules. MPNs have many biological functions, such as antioxidant and anti-bacterial, by virtue of their dynamically adjustable structural characteristics. When macromolecules such as polysaccharides are used as loading matrices, they show excellent performance beyond a single component in various environments (such as alkaline) [9,10,11]. Tea polyphenols (TP) are one of the most frequently used phenolic compounds in MPNs, as they contain many phenolic hydroxyl groups and are a common natural antioxidant. However, when TP is used alone in alkaline conditions, phenolic hydroxyl groups are ionized in large quantities, and its ability to scavenge free radicals drops sharply [12]. Magnesium oxide (MgO) has attracted considerable attention due to its green synthetic route, low toxicity, and role as a supplementary source of trace elements. In particular, MgO has a significant inhibitory effect on the main spoilage bacteria (*Enterococcus faecalis*) in tofu [13,14]. MgO can thus be considered a potential antibacterial substance for coated tofu. However, pure MPNs typically suffer from inadequate mechanical strength and limited stability. To address these limitations, biopolymers—including polysaccharides and proteins—are incorporated into MPNs to impart additional functionalities. These include enhancing mechanical strength, thermal stability, barrier performance, antibacterial and antioxidant capabilities [15]. Soluble soybean polysaccharides (SSPS) have excellent adhesion and film-forming properties [16]. Studies have shown that polysaccharide-polyphenol [17] and polysaccharide-metal ion [18,19] can form structurally stable complexes through molecular self-assembly in alkaline environments, which work in concert to improve biological activity and structural stability.

Numerous studies show that the coordination modes of MPNs are regulated by environmental pH [15]. The coordination mode of MPNs is sensitive to pH. Under strongly acidic conditions (pH < 2), monodentate binding is observed. In contrast, within the weakly acidic range (3 < pH < 6), bidentate coordination becomes the dominant form. When the environment turns alkaline (pH > 7), a shift toward tridentate binding occurs, accompanied by enhanced structural robustness as more coordination sites become involved. In view of the advantages of tridentate ligands, MPNs (SSPS-TP-MgO) were constructed and shown to have a good antioxidant effect on alkaline coated tofu [20], although its mechanism is still unclear.

This study aimed to investigate the effects of MPNs on retarding quality deterioration including texture and color, as well as on protein oxidation, protein conformation and gel microstructure of coated tofu during storage at 4 °C (simulating constant-temperature storage in household refrigerators) and 10 °C (simulating temperature fluctuations during cold-chain logistics). Pearson correlation analysis was performed to clarify the preservation efficacy of MPNs in coated tofu under alkaline conditions and reveal the intrinsic relationship between their retarding effects on quality deterioration and inhibition of protein oxidation. The findings of this study can provide strategic references for the preservation of alkaline high-protein foods.

## 2. Results and Discussion

### 2.1. Changes in the Textural Properties

**Figure 1:**
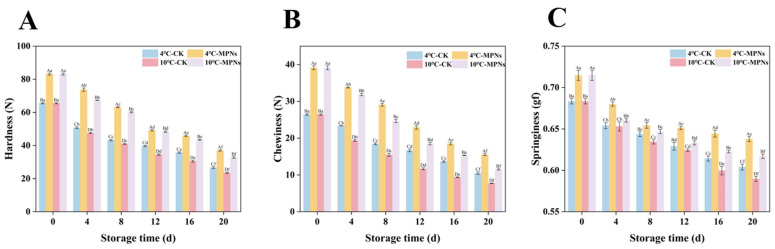
The impact of various treatments on the textural properties of coated tofu. (**A**) Hardness, (**B**) chewiness, and (**C**) springiness. The average of three independent replicates (*n* = 3) was used for each data point. Lowercase letters (a–f) show significant changes among refrigeration times (*p* < 0.05) for the same treatment, whereas uppercase letters (A–D) indicate significant differences among treatments (*p* < 0.05). 4 °C-CK: untreated samples at 4 °C. 4 °C-MPNs: samples treated with MPNs at 4 °C. 10 °C-CK: untreated samples at 10 °C. 10 °C-MPNs: samples treated with MPNs at 10 °C.

Texture is an important index to determine the structural state of coated tofu from a macroscopic perspective. Here, hardness refers to the strength required for tofu deformation; chewiness refers to the ability of tofu to resist continuous chewing; springiness refers to the ratio of recovered height following an initial compression—that is, the recovered height divided by the initial height. As shown in Figure 1, prolonged refrigeration of coated tofu resulted in a marked reduction in hardness, chewiness, and springiness (*p* < 0.05). This observation aligns with the results reported by Cui et al. [21] concerning the quality variations in packaged tofu stored under different temperature conditions. In contrast, the texture properties of the MPNs groups remained consistently superior to those of the CK group over the entire storage duration, indicating that MPNs effectively delayed the decline of textural properties. This may be due to the fact that under alkaline conditions, the phenolic hydroxyl groups in MPNs (especially the o-dihydroxy structures) are easily oxidized to o-quinones and other quinone intermediates. The generated quinones, due to strong electrophilicity, can react with nucleophilic residues in proteins via Michael addition or Schiff base formation. These reactions primarily involve cysteine sulfhydryl groups, lysine and arginine amino groups, and histidine imidazolyl groups, leading to the formation of non-disulfide covalent bonds such as C-S and C-N cross-links [22]. These interactions help stabilize the peptide structure, reducing cross-link disruption or breakage, thereby enhancing the stability of the protein-polyphenol complex and preserving the gel network’s structural integrity. Similarly, Sun et al. [23] reported that alkaline polyphenols could induce a more compact gel network in egg white protein, leading to significant improvements in texture properties (hardness, chewiness, and springiness). The texture characteristics of coated tofu at 10 °C were significantly lower than those at 4 °C, likely because cold storage at 10 °C increases the water activity of coated tofu [24], thereby decreasing its textural properties.

### 2.2. Changes in the Colour

**Figure 2:**
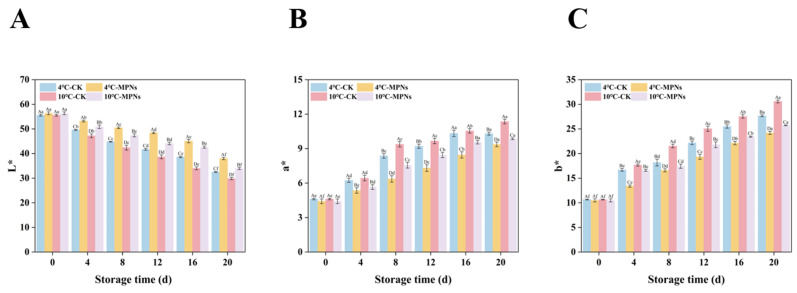
The effects of various treatments on the colour of coated tofu’. (**A**) L*, (**B**) a*, and (**C**) b*. L*: lightness, a*: greenness–redness, b*: blueness–yellowness. The average of three independent replicates (*n* = 3) was used for each data point. Lowercase letters (a–f) show significant changes among refrigeration times (*p* < 0.05) for the same treatment, whereas uppercase letters (A–D) indicate significant differences among treatments (*p* < 0.05). 4 °C-CK: untreated samples at 4 °C. 4 °C-MPNs: samples treated with MPNs at 4 °C. 10 °C-CK: untreated samples at 10 °C. 10 °C-MPNs: samples treated with MPNs at 10 °C.

Colour change is a key sensory index that influences consumers’ purchase decisions. This study used a colour difference analysis to systematically compare the variations in L* values, a* values, and b* values of coated tofu at various refrigeration temperatures. It is important to note that the sensory qualities of coated tofu were unaffected by MPNs therapy. The surface appearance of coated tofu after immersion in MPNs showed no significant differences when compared to that of untreated samples. Nevertheless, objective colour measurements revealed significant changes during storage: L* values exhibited a statistically significant downward trend over time, whereas there was a notable rise in the a* and b* values (*p* < 0.05). This change primarily stems from two mechanistic aspects: on the one hand, the Maillard reaction occurring during refrigeration generates dark-coloured substances, causing the L* value to significantly decline; on the other hand, oxidation reactions induce modifications of specific amino acid residues (e.g., tyrosine, tryptophan), forming chromophores such as dityrosine and kynurenine, which promote gradual yellowing of the product’s surface [25]. As seen in Figure 2, in the CK group, the L* value dropped to a minimum of 32.52 and 29.82 under 4 °C and 10 °C storage conditions, respectively. In contrast, in the MPNs group, the minimum L* values remained at 37.98 and 33.91, respectively. This indicates that the MPNs effectively delayed the decline in L* values, thereby maintaining the colour stability of the coated tofu. This is likely because MPNs can inhibit the progression of oxidation reactions and reduce water evaporation from the sample’s surface, keeping the coated tofu in a consistently moist state. A smooth, moist surface helps enhance visual brightness. It is noteworthy that within the same treatment group, samples stored at 4 °C consistently had greater L* values than samples stored at 10 °C. Therefore, low-temperature storage combined with MPNs can more effectively delay the oxidation and browning of coated tofu, thereby better preserving its appearance and colour, thereby improving consumer appeal.

### 2.3. Changes in the Oxidation Characteristics of Proteins

**Figure 3:**
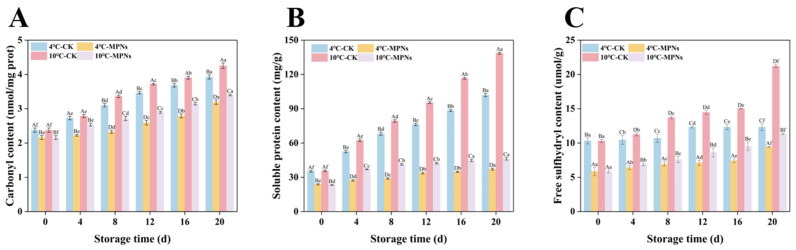
The impact of various treatments on the protein oxidation of coated tofu. (**A**) Carbonyl content, (**B**) soluble protein content, and (**C**) free sulfhydryl content. The average of three independent replicates (*n* = 3) were used for each data point. Lowercase letters (a–f) show significant changes among refrigeration times (*p* < 0.05) for the same treatment, whereas uppercase letters (A–D) indicate significant differences among treatments (*p* < 0.05). 4 °C-CK: untreated samples at 4 °C. 4 °C-MPNs: samples treated with MPNs at 4 °C. 10 °C-CK: untreated samples at 10 °C. 10 °C-MPNs: samples treated with MPNs at 10 °C.

The formation of carbonyls serves as an early-stage marker of oxidative modification in various amino acids, with the carbonyl content serving as a direct indicator of the degree of oxidation of proteins. As seen in Figure 3A, the carbonyl content exhibited a substantial rise as refrigeration time progressed. Reactive oxygen species (ROS)-mediated oxidation of amino acid side chains is responsible for this increase, converting susceptible residues into carbonyl derivatives during cold storage [26]. On day 20, the carbonyl contents in the MPNs groups reached 3.19 nmol/g at 4 °C and 3.40 nmol/g at 10 °C, representing reductions of 18.8% and 20.19%, respectively, compared with the CK group. These findings showed that MPNs preserve the structural integrity of proteins while substantially mitigating oxidative progression. The underlying mechanism lies in the ability of MPNs to scavenge free radicals, thereby protecting amino acid side chains of proteins and delaying protein oxidation and degradation in coated tofu [27]. This protective action helps maintain the structural integrity of coated tofu. Furthermore, this finding aligns well with the texture results: oxidation of proteins in coated tofu compromises the integrity of the gel network, ultimately causing the textural qualities to deteriorate. Walayat et al. [28] have previously reported that increased carbonyl content could impair the functional, structural, and gelling properties of grass fish.

Soluble protein concentration is a vital measure of protein denaturation during cold storage. As illustrated in Figure 3B, soluble protein content exhibited an increasing trend with prolonged cold storage across all sample groups, likely due to disintegration of the protein gel network and dissociation of large protein aggregates into soluble low-molecular-weight fragments. On day 20, the soluble protein content of the MPNs group at 4 °C and 10 °C was 37.2 mg/g and 46.2 mg/g, respectively, which decreased by 63.55% and 66.65% in comparison to the CK group (*p* < 0.05). These results showed that MPNs inhibited the oxidation and aggregation of proteins, efficiently postponing the decline of the gel network structure, and enhancing the prolonged stability of the protein [28]. The associated mechanism is likely that MPNs have excellent antioxidant capacity, affinity, and modification abilities at the protein interface [29], thus strengthening the coated tofu’s gel network structure through surface interactions. Wu et al. [30] showed that carbonyl and sulfhydryl content may be negatively correlated during frozen storage. The formation of carbonyl groups can facilitate the oxidation of amino acid residues and alter protein structures, which will lead to the destruction of the gel network of coated tofu and eventually increase its soluble protein content.

Free sulfhydryl concentration is a crucial predictor of the oxidation level in coated tofu during cold storage. As seen in Figure 3C, the concentration of free sulfhydryl groups in each experimental group demonstrated a substantial upward trend during cold storage (*p* < 0.05). This is likely due to the degradation and spatial conformation unfolding process of protein molecules. This alteration was attributed to the oxygen-induced conformational expansion of the protein structure, which exposed the groups that were initially embedded within the molecule, which led to the increase in free sulfhydryl group content [31]. On day 20, the free sulfhydryl concentration in the 4 °C and 10 °C MPNs groups was 9.44 μmol/g and 11.33 μmol/g, respectively, which, when compared to the CK group, dropped by 23.38% and 46.66%, respectively (*p* < 0.05). This is likely because MPNs inhibited the oxygen-mediated protein conformational unfolding and the transformation of disulfide bonds into free sulfhydryl groups, leading to a decrease in the content of free sulfhydryl groups in the MPNs groups [32]. Moreover, after 20 days at these treatment conditions, the free sulfhydryl concentration of samples stored at 10 °C was markedly higher than that of samples stored at 4 °C. This is likely because 10 °C accelerated the conversion rate of disulfide bond content into free sulfhydryl, which may further expand the gel network’s structure, and ultimately result in the augmentation of free sulfhydryl groups [33].

### 2.4. Changes in the Intermolecular Forces

**Figure 4:**
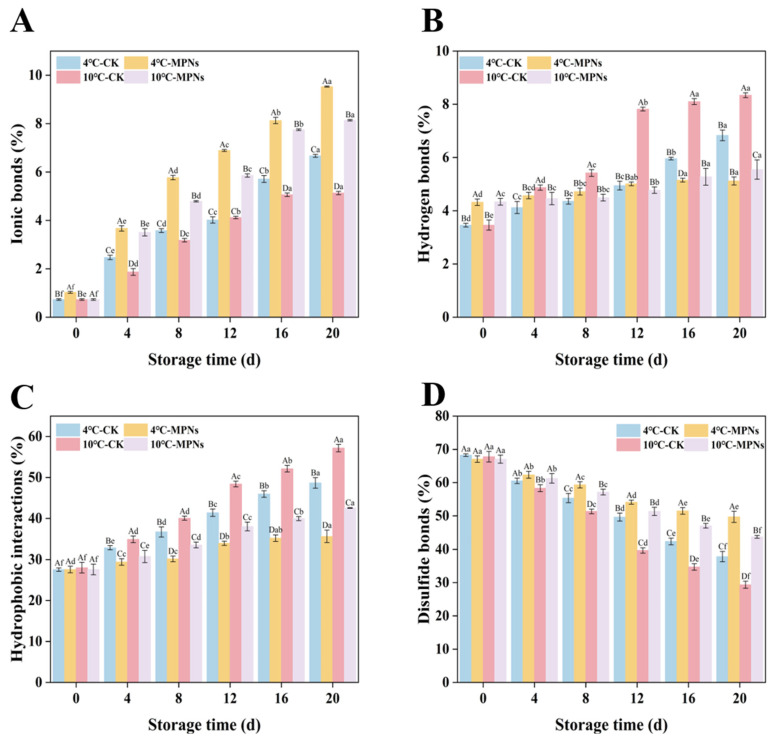
Intermolecular forces in coated tofu are affected by several treatment groups. (**A**) Ionic bonds, (**B**) hydrogen bonds, (**C**) hydrophobic interactions, and (**D**) disulfide bonds. The average of three independent replicates (*n* = 3) was used for each data point. Lowercase letters (a–f) show significant changes among refrigeration times (*p* < 0.05) for the same treatment, whereas uppercase letters (A–D) indicate significant differences among treatments (*p* < 0.05). 4 °C-CK: untreated samples at 4 °C. 4 °C-MPNs: samples treated with MPNs at 4 °C. 10 °C-CK: untreated samples at 10 °C. 10 °C-MPNs: samples treated with MPNs at 10 °C.

The intermolecular force can be used to analyse the evolution process of the gel’s three-dimensional network structures in coated tofu during refrigeration, in order to assess its structural and functional characteristics. The solubility of coated tofu in different solvents was measured to explore the intramolecular and intermolecular interaction mechanisms of proteins. Figure 4 depicts variations in the interaction forces of coated tofu during cold storage. In the intermolecular interactions, disulfide bonds and hydrophobic interactions were dominant, followed by hydrogen bonds and ionic bonds. With the extension of cold storage time, hydrophobic interactions, hydrogen bonds, and ionic bonds increased (*p* < 0.05), while disulfide bonds decreased. Disulphide bonds are essential to preserve protein structures [34], and their decrease indicates that the three-dimensional gel network structure of coated tofu is damaged. In this instance, the protein structure is opened, and hidden hydrophobic groups are exposed, thus increasing the hydrophobic interactions of coated tofu [35]. Moreover, the aggregation and crosslinking of coated tofu’s gel structure may improve ionic and hydrogen bonds [36]. Relative to the CK group, the intermolecular forces of the MPNs groups changed less, mainly because MPNs can form a variety of weak interactions through noncovalent bonds to enhance the stability of proteins [37].

### 2.5. Changes in the Secondary Structures

**Figure 5:**
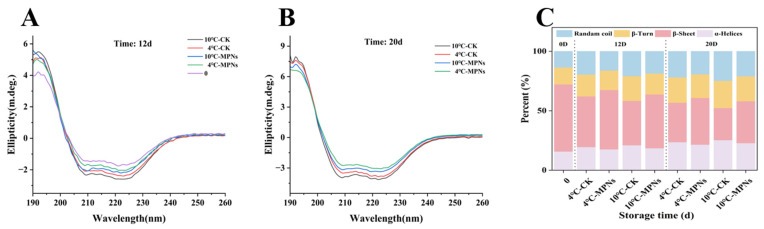
The impact of several treatment groups on the secondary structures of coated tofu. (**A**) Circular dichroism on the 12th day, (**B**) circular dichroism on the 20th day, and (**C**) secondary structure proportion. 4 °C-CK: untreated samples at 4 °C. 4 °C-MPNs: samples treated with MPNs at 4 °C. 10 °C-CK: untreated samples at 10 °C. 10 °C-MPNs: samples treated with MPNs at 10 °C.

The secondary structures of proteins are an important index to reflect the conformational changes in coated tofu. As can be seen from Figure 5, a characteristic band signal can be observed by analysing the circular dichroism spectrum of the samples. Among these, α -helix structures show an optimistic band around 192 nm, and two typical negative bands at 208 nm and 222 nm. Moreover, β-sheet structures present a positive band at 190 nm, and an obvious negative characteristic band near 215 nm. The positions and directions of these bands are consistent with the standard spectral characteristics of the corresponding secondary structures in proteins.

As can be seen from Figure 5, when the cold storage time was prolonged, the number of random coils, β-turns, and α-helices increased while the fraction of β-sheets dropped, indicating that the protein structure gradually changed from an ordered β-sheet to a disordered conformation, which is consistent with Xie et al.’s study on the conformational changes in coated tofu during heat treatment [2]. Specifically, after 20 days, the β-sheet content of the MPNs groups at 4 °C and 10 °C decreased by 15.26% and 19.28%, which, at 10 °C, was significantly lower than that of the CK group, with decreases of 21.32% and 27.65%, respectively. This showed that MPNs can delay conformational changes in coated tofu, likely because MPNs self-assemble quickly on the surface of coated tofu under alkaline conditions, forming an effective physical barrier layer that reduces its contact area with oxygen in the air [38]. In addition, the molecular coordination effect between TP and MgO enhanced the compactness of the coating, which together slowed down the decomposition rate of proteins. Interestingly, the β-sheet content was higher in samples stored at 4 °C than in those stored at 10 °C, which is likely due to the fact that the stability of protein structures can be better maintained at lower temperatures. On the other hand, higher temperatures (10 °C) likely weaken this stability, making it easier for the protein gel network to grow and the interior hydrophobic group to become apparent. The enhancement of hydrophobic interactions subsequently increases the collision probability of protein granules, which enhance the degree of damage to the secondary structures.

### 2.6. Changes in the Endogenous Fluorescent Spectrometry

**Figure 6:**
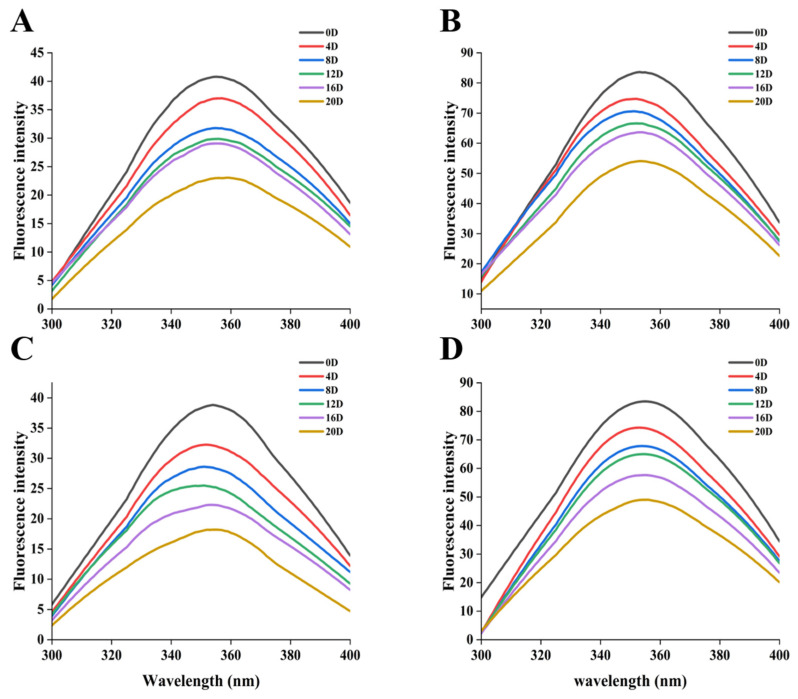
Influence of different treatments on the fluorescence spectrum of coated tofu. (**A**) 4 °C-CK, (**B**) 4 °C-MPNs, (**C**) 10 °C-CK, and (**D**) 10 °C-MPNs. 4 °C-CK: untreated samples at 4 °C. 4 °C-MPNs: samples treated with MPNs at 4 °C. 10 °C-CK: untreated samples at 10 °C. 10 °C-MPNs: samples treated with MPNs at 10 °C.

Endogenous fluorescence spectroscopy is a useful tool to characterise the shifting of protein tertiary structures in coated tofu. A drop in fluorescence intensity indicates cross-linking and protein aggregation. As shown in Figure 6, the fluorescent intensity of samples exhibited a continuous declining trend with prolonged refrigeration time. This phenomenon arises from fluorescence quenching induced by the disruption of protein conformations. These structural alterations cause the gel network to unfold, which makes it easier for internal hydrophobic groups to be exposed to a polar milieu [39]. When the refrigeration period ends (day 20), the fluorescent intensity of the MPNs groups (especially under 4 °C conditions) is considerably higher than the CK groups. This phenomenon might be connected to TP’s cross-linking ability: after conformational disruption of proteins, hydrophobic groups become exposed. TP, acting as a protein cross-linker, can promote the aggregation of the gel structure [40], thereby mitigating the decrease in fluorescence intensity caused by alterations in the hydrophobic microenvironment. Compared to 4 °C, the fluorescence peak intensity at 10 °C was noticeably higher, indicating that lower-temperature refrigeration has a stabilizing effect on the tertiary structures of proteins. The temperature-dependent acceleration of protein oxidation rates is likely the cause of this discrepancy: a higher temperature (10 °C) accelerates oxidation, intensifies conformational fluctuations in tertiary structures, and leads to a loss of stability, which in turn causes buried tryptophan residues to become more exposed to a polar microenvironment and consequently results in a notable decrease in fluorescence intensity [41]. This observation is consistent with the secondary structure results, in which the shift from ordered to disordered structures further exposes hydrophobic groups and increases the tryptophan microenvironment’s polarity, ultimately compromising the stability of the protein tertiary structures.

### 2.7. Changes in the SDS-PAGE

**Figure 7:**
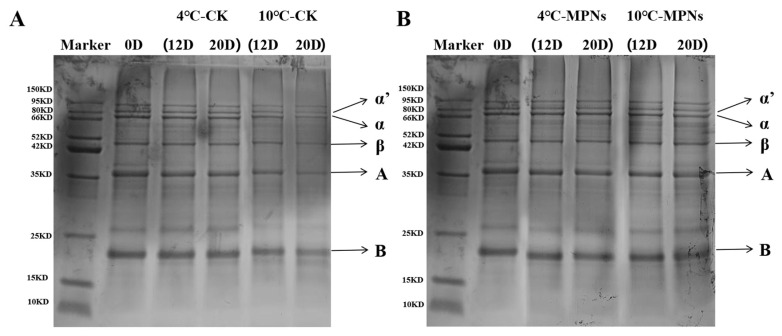
The impact of different treatment groups on the SDS-PAGE profile of coated tofu. (**A**) untreated samples, (**B**) samples treated with MPNs.

To investigate the degradation or aggregation behaviour of proteins during refrigeration, the molecular weight distribution of the proteins in coated tofu was investigated using SDS-PAGE. As shown in Figure 7, electrophoretic analysis identified 7S (α’, α, and β subunits) and 11S (A and B subunits) globulins as the major protein components found in coated tofu. With prolonged refrigeration time, all protein fractions exhibited varying degrees of degradation, with some bands weakening or even disappearing completely, indicating that the proteins in coated tofu were oxidized during refrigeration, and the degradation of large molecules resulted in the formation of small proteins and peptides [42]. This outcome is in line with the observed variations in soluble protein concentration. Furthermore, the gel network structure that preserves the texture of coated tofu was disrupted as a result of the oxidative degradation of proteins [43], consequently resulting in a decline in hardness, chewiness, and springiness. These findings are consistent with our measurements of textural properties. On day 20, the band intensity of proteins in the CK group was markedly reduced, with more pronounced degradation observed at 10 °C. Specifically, in the 10 °C CK group (Table 1), the content of the a′, a, and A subunits declined by 2.92%, 2.13%, and 6.24%, respectively. In contrast, the degradation in the 10 °C coated group was less pronounced, with the band intensities of the α’, α, and A subunits decreasing by only 1.95%, 1.50%, and 0.17%, respectively. This indicates that MPNs effectively mitigated the degradation of the α’, α, and A subunit contents, resulting in a protein band distribution closer to that observed at the initial refrigeration stage. This protective effect stems from the ROS-responsive properties of MPNs: the phenolic hydroxyl groups can provide reactive hydrogen atoms, effectively neutralizing ROS [44], thereby mitigating oxidative damage to proteins and preserving their structural integrity.

### 2.8. Changes in the Scanning Electron Microscopy (SEM)

**Figure 8:**
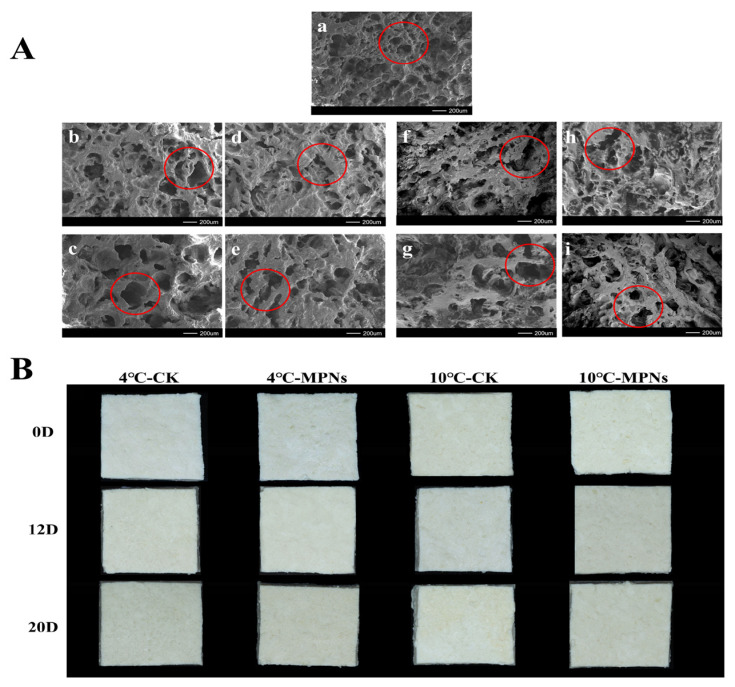
Microstructural and morphological features of coated tofu influenced by various treatment groups. (**A**) Microstructure, and (**B**) appearance. (**a**) 0D, (**b**) 4 °C-CK-12D, (**c**) 4 °C-CK-20D, (**d**) 4 °C-MPNs-12D, (**e**) 4 °C-MPNs-20D, (**f**) 10 °C-CK-12D, (**g**) 10 °C-CK-20D, (**h**) 10 °C-MPNs-12D, and (**i**) 10 °C-MPNs-20D. 4 °C-CK: untreated samples at 4 °C. 4 °C-MPNs: samples treated with MPNs at 4 °C. 10 °C-CK: untreated samples at 10 °C. 10 °C-MPNs: samples treated with MPNs at 10 °C.

SEM and the appearance (Figure 8) of coated tofu under different treatment conditions reveal that the microstructure of coated tofu was closely correlated with its quality attributes. At the start of the refrigeration period (day 0), the surface morphology of the coated tofu appeared smooth and fine-textured, exhibiting a uniform and dense three-dimensional network structure. Its fine porous structure contributed to an enhanced water-holding capacity, thereby imparting favourable protein gel hardness and elasticity to the product [45]. During refrigeration, the microstructure of the coated tofu underwent significant changes (*p* < 0.05): the spacing between protein chains increased, the network gradually loosened and became disordered, and the surface roughness was enhanced. After 20 days of refrigeration, the CK group of coated tofu exhibited marked quality deterioration. At 4 °C, the gel network structure was disrupted, forming a typical honeycomb-like porous structure; meanwhile, samples stored at 10 °C exhibited surface yellowing and a soft, mushy texture. These observations visually reflect the extent of microstructural damage caused by protein oxidation and unfolding. In contrast, the MPNs group maintained a dense and uniform microstructure, confirming its positive role in stabilizing the protein network. This is because MPNs may induce the directed aggregation of proteins through hydrogen bonding and covalent interactions under alkaline conditions, thereby constructing a gel system with enhanced structural stability [46].

### 2.9. Correlative Analysis

**Figure 9:**
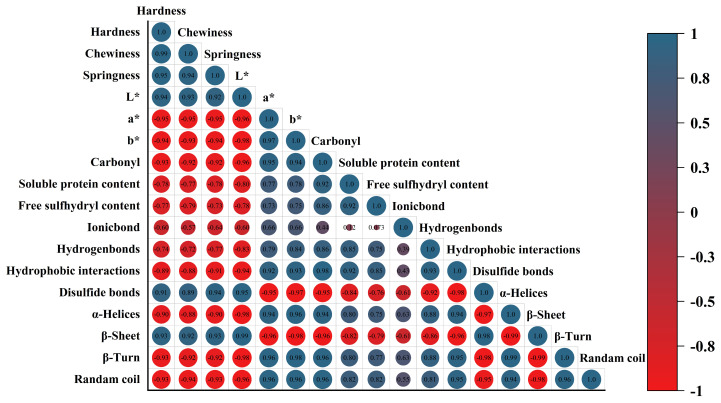
Correlation analysis of protein aggregation characteristics. (L*: lightness, a*: greenness–redness, b*: blueness–yellowness).

This study systematically investigated correlations among the quality, protein oxidation, and structure of coated tofu during refrigeration using a Pearson correlation analysis. As shown in Figure 9, β-sheet content was shown to be strongly correlated with hardness, chewiness, and springiness (*p* < 0.01), suggesting that β-sheet structures are essential to preserve the textural characteristics of tofu. Meanwhile, L* value showed significant negative correlations with carbonyl content, α-helices, β-turns, and random coils (*p* < 0.01), suggesting that protein oxidation and the resulting conformational changes were associated with the decrease in sample brightness. This may be attributed to light scattering effects caused by the Maillard reaction or protein aggregation. Carbonyl content showed significant positive correlations with soluble protein concentration, free sulfhydryl content, hydrogen bonds, hydrophobic interactions, α-helices, β-turns, and random coils (*p* < 0.01), indicating that during refrigeration, the accumulation of ROS led to the oxidative conversion of side-chain amino groups to carbonyl compounds. However, the oxidative environment promoted the breakage or rearrangement of disulfide bonds within or between proteins, contributing to a marked increase in free sulfhydryl content. At the same time, protein polymers were oxidatively degraded into small soluble fragments, thereby increasing the soluble protein content. In addition, oxidation compromised the integrity of hydrogen bonds and hydrophobic interactions that maintain structural stability, promoting the breakdown of protein secondary structures and further disrupting the gel network. On the other hand, carbonyl content showed significant negative correlations with textural properties and β-sheet content (*p* < 0.01), indicating that protein oxidation led to the accumulation of carbonyl compounds, which led to a decrease in β-sheet structures that maintain the ordered conformation of proteins, a decrease in protein spatial structure stability, and a decrease in intermolecular interactions and gel support ability, leading to a significant decrease in the textural properties of coated tofu. In summary, protein oxidation served as the primary driver of texture deterioration and structural changes in coated tofu. It destabilized hydrogen bonds, hydrophobic interactions, and disulfide bonds, facilitating the shift from ordered to disordered secondary structures and consequently leading to a decline in product quality.

## 3. Conclusions

In summary, this study demonstrates that metal-phenolic networks (MPNs) effectively inhibit protein oxidation, delay quality deterioration, and preserve the structural integrity of coated tofu under alkaline cold storage conditions. Specifically, MPNs treatment significantly reduced protein degradation, maintained secondary structure stability and gel microstructure integrity, and stabilized the 11S protein subunits, thereby contributing to an extended shelf life. Furthermore, correlation analysis revealed that the preservative effect of MPNs is closely associated with the regulation of protein conformation and disulfide bond stability.

Despite these findings, the precise mechanisms by which MPNs delay quality deterioration and mitigate protein oxidation in coated tofu remain to be fully elucidated. Additionally, comparative studies involving commonly used antioxidants, such as tea polyphenols, are necessary to contextualize the efficacy of MPNs relative to existing preservation strategies. Moreover, the present study did not evaluate changes in free amino acid composition, which is important for assessing nutritional quality. Similarly, lipid oxidation markers were not monitored, given the relatively low lipid content of coated tofu. Such investigations will facilitate a more robust understanding of MPNs-based interventions and support their potential application in the preservation of alkaline high-protein gel foods.

## 4. Materials and Methods

### 4.1. Materials and Chemicals

Coated tofu samples (3 × 3 × 1 cm) were prepared and provided by the Hunan Provincial Key Laboratory of Soybean Products Processing and Safety (Shaoyang, China). Soluble soybean polysaccharides (purity ≥ 99%) were sourced from Linyi Shansong Biological Products Co., Ltd. (Linyi, China). TP (purity ≥ 99%), MgO (purity ≥ 99%), and L-(-)-Malic acid (purity ≥ 98%) were obtained from Shanghai Macklin Biochemical Co., Ltd. (Shanghai, China). Every other chemical used was analytically pure.

### 4.2. Preparation of Composite Preservatives

The successful preparation of the MPNs was confirmed by ultraviolet-visible (UV-Vis) absorption spectroscopy (UV-1800; Shimadzu Corporation; Kyoto, Kyoto Prefecture, Japan), scanning electron microscopy (SEM) (TESCAN MIRA LMS, Brno, Czech Republic), and energy-dispersive X-ray (EDX) elemental mapping analysis. As described in the following reference [20], the concentration of MPNs and the soaking time for coated tofu were optimized through extensive preliminary experiments, yielding free radical scavenging activities of 66.70 ± 0.89% for ABTS and 60.94 ± 0.48% for DPPH. A total of 0.26 g of MgO was added to 1.56 mL (5 mol/L) of L-(-)-Malic acid solution and dissolved in 40 mL of sterile water. Next, 2.1 g of TP and 0.02 g of SSPS were added and stirred by magnetic force for 0.5 h. The solution’s pH was brought to 8.5 using sodium hydroxide (consistent with the pH of coated tofu), and the mixture was diluted to 50 mL using sterile water to produce MPNs (SSPS-TP-MgO). The CK group was treated with sterile saline.

### 4.3. Preparation of Coated Tofu

The tofu was trimmed into 3 cm × 3 cm × 1 cm uniform blocks and immersed in a 2% sodium bicarbonate solution and a 0.7% sodium chloride solution for 10 h to make coated tofu (pH of 8.5). Subsequently, the coated tofu samples were submerged in MPNs for ten minutes (MPNs groups). For the same duration, the CK samples (CK groups) were submerged in sterile water. After soaking, samples were placed on a sterile surface, and moisture was gently removed using sterile filter paper. The samples were then packed in sealed aseptic packaging containers. Samples from the MPNs group and CK group were randomly divided into two groups and stored in a constant temperature incubator at either 4 °C or 10 °C. After storage, various indexes were determined and analysed.

### 4.4. Textural Properties

Texture profiles were analysed using a method described by Huang et al. [47] with minor modifications and a texture analyser (LS-5, AMETEK, Inc., Berwyn, IL, USA). Measurements were taken at five locations (four corners and the centre) for each sample with three replicates per location. A P/35 cylindrical flat probe was used under the following conditions: 40 mm/s prior to the test, 30 mm/s during the test, and 40 mm/s after the test. The trigger force was set to 0.05 N with a 5-s interval between measurements. Hardness, springiness, and chewiness were evaluated, with five repeated measurements conducted per sample.

### 4.5. Colour

A CR-400 colorimeter (Konica Minolta, Kyoto, Japan) was used to measure colour. Before use, the instrument was calibrated using a white reference plate under the CIE standard illuminant D65 (Konica Minolta, Kyoto, Japan). Colour measurements were carried out using a d/8° optical geometry (diffuse illumination/8° directed viewing) with a circular measuring aperture of 8 mm in diameter. Measurements were taken at six areas along the diagonal of the coated tofu surface, and L*, a*, and b* values were calculated.

### 4.6. Oxidation Characteristics of Proteins

A slightly modified version of the method described by Xu et al. [48] was used to measure protein solubility. A total of 1 g of coated tofu was weighed and added to 9 mL of phosphate buffer (PBS, 0.01 mol/L, pH 8.0). The combination was homogenised in a high-speed homogeniser for two minutes at 12,000 rpm until the tofu was completely dissociated. The supernatant was collected after the homogenate was centrifuged for 20 min at 4000 *g*. The BCA method was used to determine the amount of soluble protein in the supernatant. A microplate reader (Epoch, BioTek Instruments, Winooski, VT, USA) was used to detect absorbance at 562 nm. Reagents A and B were combined at a 50:1 ratio to create the BCA working solution. To create a standard curve spanning from 0 to 500 μg/mL, aliquots of 0–20 μL of protein standard solution were diluted to a final volume of 20 μL. The absorbance was determined after 20 μL of the test protein and 200 μL of the working solution were combined and incubated at 37 °C for 20–30 min. The standard curve was used to then calculate the protein concentrations of samples.

The determination of carbonyl content according to the method of Zhu [19] with minor modification. After repeating the steps described in 4.6, the soluble proteins of supernatants were diluted to a 0.5 mg/mL concentration. 1 mL of supernatants (0.5 mg/mL) was mixed with 1 mL DNPH solution (10 mM), incubated in the dark for 1 h. Afterward, 1 mL trichloroacetic acid (TCA, 20%, *w*/*w*) was added to the mixture, followed by precipitation and centrifugation (12,000 *g*, 10 min, 4 °C), then washed with ethanol: ethyl acetate solution (1:1, *v*/*v*) three times. The pellets were dissolved in 6 M guanidine hydrochloride. The supernatant absorbance was measured at 370 nm

With a few minor adjustments, the free sulfhydryl concentration was calculated using the method described by Xue et al. [15]. Following the crushing of coated tofu, 3 g of tofu was weighed, diluted in 27 mL of PBS (0.01 mol/L, pH 8.0) buffer, and centrifuged at 4 °C for 20 min at 4000 *g*. After collecting the supernatant, the BCA method was used to determine the protein content. Here, 2.8 mL of Tris-Gly buffer, 0.02 mL of Ellman’s reagent (4 mg/mL DTNB solution made in Tris-glycine buffer), and 0.2 mL of the supernatant were combined. A UV-Vis spectrophotometer (UV-1800; Shimadzu Corporation; Kyoto, Kyoto Prefecture, Japan) was used to measure the absorbance at 412 nm after the reaction was thoroughly mixed and left in the dark for 15 min. The free sulfhydryl content was calculated using the following formula:

(1) $$\mathrm{SH}(\mu \mathrm{mol}/L)\;=\;73{.53\;\times \;A}_{412}\;\times \;\frac{D}{C}$$

where A_412_ is the observed absorbance at 412 nm, D is the dilution multiple, C is the protein concentration of the sample supernatant, and 73.53 is a constant obtained from the computation of 10^6^/(1.36 × 10^4^).

With a few minor adjustments, intermolecular forces were measured using the methodology outlined by Liu et al. [28]. A total of 10 mL of four distinct solutions were mixed with five aliquots (1 g each) of each coated tofu sample: 0.6 mol/L NaCl (S1); 0.6 mol/L NaCl with 1.5 mol/L urea (S2); 0.6 mol/L NaCl with 8.0 mol/L urea (S3); and 0.6 mol/L NaCl with 8.0 mol/L urea and 0.2 mol/L β-mercaptoethanol (S4). Following complete homogenisation, 50 mL of volume was added, and the sample was centrifuged for 10 min at 12,000 *g*. After collecting the supernatant, a BCA kit was used to test the protein concentration (Biotechnology Co., Ltd., Nanjing, China). The following formula (S1, S2, S3, and S4 are replicates) was used to determine the relative concentration of ionic bonds, hydrogen bonds, hydrophobic interactions, and disulphide bonds:

S1 (%) = [Protein concentration in S1/(Total protein concentration in S1 + S2 + S3 + S4)] × 100%(2)

### 4.7. Secondary Structure Analysis

After repeating the steps described in 4.6, the soluble proteins of supernatants were diluted to a 0.5 mg/mL concentration. With a bandwidth of 1 nm, a reaction time of 0.5 s, and a spectral measuring speed of 50 nm/min, a 0.1 mm quartz colorimetric utensil was used to measure spectra at 25 °C from 190 to 260 nm. The secondary structure contents were then examined using a circular dichroism spectrometer (Applied Photophysics Ltd., Leatherhead, UK) equipped with the Circular Dichroism Neural Network (CDNN) software(CDNN 2.1).

### 4.8. Endogenous Fluorescence Spectroscopy

After repeating the steps described in 4.6, the soluble proteins of supernatants were diluted in PBS buffer (0.01 mol/L, pH 8.0) to a concentration of 0.5 mg/mL. Using a fluorescence spectrometer (FLS1000, Edinburgh, UK), the detection settings were 290 nm for the excitation wavelength, 300–400 nm for the emission scanning range, and 5 nm for the slit width [49]. Each measurement was performed three times.

### 4.9. SDS-SPAGE

With a few minor adjustments, SDS-PAGE was carried out using a technique described by Pan et al. [50]. In short, each specimen (1 g) was carefully weighed, combined with 9 mL of a 5% sodium dodecyl sulphate (SDS) solution, and heated to 85 °C for 1.5 h. After cooling, and following centrifugation for ten minutes at 7200 *g*, the supernatant was recovered. A BCA kit (Biotechnology Co., Ltd., Nanjing, China) was used to measure the protein concentration, which was then corrected to 5 mg/mL. The supernatants were combined with reductive loading buffer at a volume ratio of 4:1 to create reduced samples. The reduced samples were thoroughly combined, heated in a boiling water bath for five minutes, rapidly cooled to room temperature, and then centrifuged at 7200 *g* for five minutes. Subsequently, 8 μL of supernatant was collected for gel loading. When the samples reached the interface between the 5% stacking gel and the 12% resolving gel, electrophoresis was performed at a constant voltage of 80 V for approximately an hour. Next, the voltage was changed to 120 V and kept constant until the gel reached the dye front. After the experiment concluded, the gel was stained with staining solution at room temperature for 1 h, followed by decolorizing (approximately 24 h) until the background became clear, and results were recorded.

### 4.10. Scanning Electron Microscope (SEM)

With a few minor adjustments, scanning electron microscopy (SEM) was performed using the method outlined by Cai et al. [51]. Briefly, the coated tofu samples were divided into roughly 0.5 × 0.5 × 0.5 cm^3^ cubes and then fixed in a 2.5% glutaraldehyde solution for two hours. Next, PBS buffer (0.1 M, pH 7.2) was used to rinse the samples five to ten times. A graded sequence of ethanol solutions (30%, 50%, 70%, 90%, and 100%) was then used for gradient dehydration, each lasting ten minutes. After air-drying at room temperature to remove volatile organic compounds, the samples were freeze-dried for 15 h. Before imaging, the samples were coated with gold using sputter deposition at 10 mA for 45 s. Surface morphology was then imaged at an accelerating voltage of 3 KV, and energy-dispersive spectroscopy was performed at 15 kV.

### 4.11. Statistical Analysis

All experimental data was statistically analysed using the IBM SPSS Statistics 23 software (IBM Corp., Armonk, NY, USA). All data are shown using the mean ± standard deviation (SD). Duncan’s test and analysis of variance (ANOVA) were used to assess treatment differences; a *p* < 0.05 was deemed significant. Additionally, the correlations between qualitative features (colour and texture), protein oxidation indices, and structural parameters of coated tofu during refrigeration were assessed using Pearson’s correlation analysis.

## Figures and Tables

**Table 1:** The impact of several treatment groups on the decreased protein band intensity of coated tofu.

Groups	α’	α	β	A	B
0D	11.13 ± 0.13 ^Aa^	13.31 ± 0.12 ^Aa^	10.35 ± 0.05 ^Aa^	18.50 ± 0.18 ^Aa^	31.21 ± 0.16 ^Ab^
4 °C-CK-12D	10.64± 0.14 ^Bb^	12.70 ± 0.11 ^Bb^	11.19 ± 0.04 ^ABb^	17.91 ± 0.17 ^Bb^	31.57 ± 0.19 ^Bb^
4 °C-MPNs-12D	11.06 ± 0.26 ^Aa^	13.08 ± 0.06 ^Ab^	10.77 ± 0.27 ^Aab^	18.49 ± 0.3 ^Aa^	31.86 ± 0.31 ^Bb^
4 °C-CK-20D	9.15 ± 0.18 ^Bc^	12.73 ± 0.13 ^Bb^	11.57 ± 0.15 ^Ab^	17.45 ± 0.2 ^Bc^	32.49 ± 0.24 ^Ca^
4 °C-MPNs-20D	10.66 ± 0.3 ^Ab^	12.89 ± 0.08 ^Ab^	11.19 ± 0.14 ^Ab^	18.46 ± 0.33 ^Ca^	32.32 ± 0.32 ^Da^
10 °C-CK-12D	8.44 ± 0.11 ^Cb^	12.40 ± 0.20 ^Ab^	11.11 ± 0.03 ^Bb^	17.95 ± 0.19 ^Bb^	37.09 ± 0.16 ^Ab^
10 °C-MPNs-12D	10.42 ± 0.36 ^Bb^	13.03 ± 0.34 ^Aa^	11.11 ± 0.05 ^Aa^	18.43 ± 0.38 ^Aa^	30.27 ± 0.4 ^Cc^
10 °C-CK-20D	8.21 ± 0.13 ^Cb^	11.18 ± 0.22 ^Cb^	11.33 ± 0.11 ^Aa^	12.26 ± 0.24 ^Cc^	38.86 ± 0.39 ^Aa^
10 °C-MPNs-20D	9.18 ± 0.33 ^Bc^	11.81 ± 0.31 ^Bb^	11.31 ± 0.13 ^Bb^	18.33 ± 0.37 ^Aa^	33.26 ± 0.24 ^Ba^

The average of three independent replicates (*n* = 3) were used for each data point. Lowercase letters (a–c) show significant changes among refrigeration times (*p* < 0.05) for the same treatment, whereas uppercase letters (A–D) indicate significant differences among treatments (*p* < 0.05). 4 °C-CK: untreated samples at 4 °C. 4 °C-MPNs: samples treated with MPNs at 4 °C. 10 °C-CK: untreated samples at 10 °C. 10 °C-MPNs: samples treated with MPNs at 10 °C.

## Data Availability

The original contributions presented in this study are included in the article. Further inquiries can be directed to the corresponding authors.
